# Does a Screening Trial for Spinal Cord Stimulation in Patients with Chronic Pain of Neuropathic Origin have Clinical Utility and Cost-Effectiveness? (TRIAL-STIM Study): study protocol for a randomised controlled trial

**DOI:** 10.1186/s13063-018-2993-9

**Published:** 2018-11-16

**Authors:** Sam Eldabe, Ashish Gulve, Simon Thomson, Ganesan Baranidharan, Rui Duarte, Susan Jowett, Harbinder Sandhu, Raymond Chadwick, Morag Brookes, Anisah Tariq, Jenny Earle, Jill Bell, Anu Kansal, Shelley Rhodes, Rod S. Taylor

**Affiliations:** 1 The James Cook University Hospital, Middlesbrough, UK; 2 Basildon and Thurrock University Hospitals, Basildon, UK; 3 Leeds Teaching Hospitals, Leeds, UK; 4 University of Liverpool, Liverpool, UK; 5 University of Birmingham, Birmingham, UK; 6 University of Exeter, Exeter, UK; 7 University of Warwick, Warwick, UK; 8 University of Teesside, Middlesbrough, UK; 9 Patient and Public Involvement Representatives, Middlesbrough, UK

**Keywords:** Randomised controlled trial, Spinal cord stimulation, Screening trial, Neuropathic pain

## Abstract

**Background:**

The TRIAL-STIM Study aims to assess the diagnostic performance, clinical outcomes and cost-effectiveness of a screening trial prior to full implantation of a spinal cord stimulation (SCS) device.

**Methods/design:**

The TRIAL-STIM Study is a superiority, parallel-group, three-centre, randomised controlled trial in patients with chronic neuropathic pain with a nested qualitative study and economic evaluation. The study will take place in three UK centres: South Tees Hospitals NHS Foundation Trust (The James Cook University Hospital); Basildon and Thurrock University Hospitals NHS Foundation Trust; and Leeds Teaching Hospitals NHS Trust. A total of 100 adults undergoing SCS implantation for the treatment of neuropathy will be included. Subjects will be recruited from the outpatient clinics of the three participating sites and randomised to undergo a screening trial prior to SCS implant or an implantation-only strategy in a 1:1 ratio. Allocation will be stratified by centre and minimised on patient age (≥ 65 or < 65 years), gender, presence of failed back surgery syndrome (or not) and use of high frequency (HF10™) (or not). The primary outcome measure is the numerical rating scale (NRS) at 6 months compared between the screening trial and implantation strategy and the implantation-only strategy. Secondary outcome measures will include diagnostic accuracy, the proportion of patients achieving at least 50% and 30% pain relief at 6 months as measured on the NRS, health-related quality-of-life (EQ-5D), function (Oswestry Disability Index), patient satisfaction (Patients’ Global Impression of Change) and complication rates. A nested qualitative study will be carried out in parallel for a total of 30 of the patients recruited in each centre (10 at each centre) to explore their views of the screening trial, implantation and overall use of the SCS device. The economic evaluation will take the form of a cost–utility analysis.

**Discussion:**

The TRIAL-STIM Study is a randomised controlled trial with a nested qualitative study and economic evaluation aiming to determine the clinical utility of screening trials of SCS as well as their cost-effectiveness. The nested qualitative study will seek to explore the patient’s view of the screening trials, implantation and overall use of SCS.

**Trial registration:**

ISRCTN, ISRCTN60778781. Registered on 15 August 2017.

**Electronic supplementary material:**

The online version of this article (10.1186/s13063-018-2993-9) contains supplementary material, which is available to authorized users.

## Background

Approximately 20% of the adult European population has significant chronic pain, and 7–8% of the population has chronic pain with neuropathic features [1–3]. Health-related quality of life is significantly poorer in people with chronic pain than in those without [4], and poorer in people with neuropathic pain than in those with non-neuropathic pain [5]. In routine clinical care, up to 50% of patients with neuropathic pain fail to obtain pain relief from analgesic medicines [6].

In 2008, the National Institute for Health and Care Excellence (NICE) recommended spinal cord stimulation (SCS) as an effective and cost-effective treatment for severe neuropathic pain refractory to medical management conditional on a screening trial being conducted before a final implant of SCS in every case [4, 7]. The evidence for effectiveness and cost-effectiveness of such SCS screening trials still remains unclear.

### SCS screening trial and purpose

An SCS screening trial consists of the insertion of a wire (lead) introduced into the epidural space of the spinal cord through a needle puncture. The lead is then positioned to target the pain by passing current into the lead from an external power source, generating paraesthesia over the painful area. Pain coverage is expressed as a percentage of the whole area of pain covered by the paraesthesia. In patients where it is possible to achieve ≥ 80% coverage, the wire can be fixed to the spinal fascia through an open procedure and tunnelled to exit through the skin away from the incision. This technique is known as a definitive trial because the same wire is used later to attach to a battery if the trial is successful [8]. In some cases the implantation of the lead may be guided by anatomical landmarks rather than patient feedback. In an alternative technique known as a temporary trial, a wire is simply fixed to the skin at the site of the epidural needle puncture of the back by sutures or tape. The wire can be removed without the need for a surgical intervention at the end of the trial but requires a repeat implantation in the case of a successful trial. Trials can last from a few minutes (‘on-table trial’) to a few weeks (‘home trial’).

The screening trial period allows the patient to test the efficacy of SCS therapy directly and specifically. An expert panel defined a successful trial as the patient reporting ≥ 50% pain relief during the trial with stable or reduced pain medications, with at least stable levels of daily activity [9].

### Evidence for SCS screening trials as a predictor of long-term success

SCS screening trials are recommended by consensus expert opinion and national guidance in many countries, including the United Kingdom. However, the research literature to date has not provided definitive evidence to support the value of screening trials for SCS and in particular their ability to reliably predict the long-term success of SCS therapy.

The PROCESS RCT trial randomised neuropathic pain of failed back surgery syndrome (FBSS) compared SCS to conventional medical management; 95% of the patients receiving SCS reported ≥ 50% pain relief at the screening trial phase, but by contrast only 48% reported the same outcome at 6-month follow-up [10]. Another RCT compared SCS to reoperation in FBSS; 19/24 (80%) subjects reported a successful screening trial, but only 9/15 (60%) continued to report ≥ 50% pain relief at the 2-year follow-up [11]. However, no pain outcome data were available for patients who failed the trial.

Similarly, the SENZA randomised trial comparing high-frequency SCS to conventional SCS reported that 90/97 (92.8%) of subjects who trialled a higher frequency of SCS and 81/92 (88.0%) subjects who were trialled with traditional SCS were eligible for implantation [12]. By 12 months the figures for ≥ 50% pain relief dropped to 78.7% and 51.3% respectively. Oakley et al. [13] showed that 55% (18/33) of patients had ≥ 50% pain relief at the 6-month follow-up compared to 75% in the trial period. Screening trials have been shown to exclude good candidates for SCS. Twelve patients who failed to obtain ≥ 50% pain relief during an SCS screening trial still received an implanted SCS device [13]. At follow-up, a third of the patients reported pain relief rates of 44–88%. The authors concluded that the screening trial is not the sole predictor of long-term success but, again, did not assess pain outcomes in trial failures.

A retrospective study compared a 15-min on-table trial to a 5-day home trial and found a 98% (53/54) success rate for 15-min trials compared to 90% (47/52) for 5-day home trials [14]. The authors concluded that since both methods appeared to have equivalent predictive value for successful long-term outcomes, home trials should be eliminated based on increased therapeutic failures, greater risk of infection and additional therapy costs.

### Hypothesis and aims

We hypothesise that a no-SCS screening trial strategy will be superior to an SCS screening trial and more cost-effective. Specific aims of this study are to:

- compare the patient-related outcomes of an SCS screening trial strategy to a no-trial implantation-only strategy;
- determine the diagnostic performance (e.g. sensitivity and specificity) of an SCS screening trial;
- compare the cost-effectiveness of a screening trial strategy to a no-test implant-only strategy; and
- assess the expectations and experiences of patients for the use of an SCS screening trial.

## Methods and design

### Study design

**Fig. 1:**
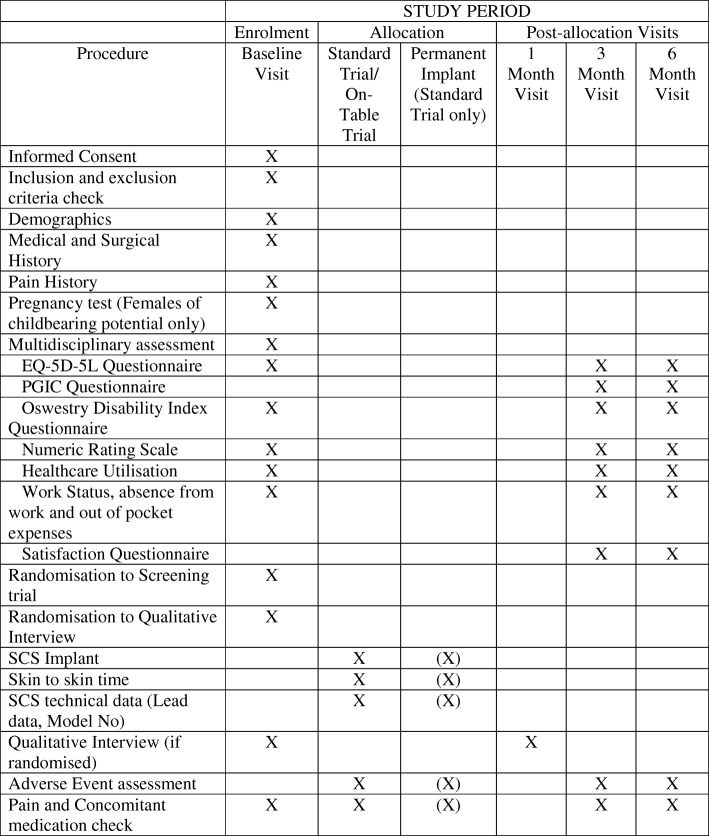
Standard Protocol Items: Recommendations for Interventional Trials (SPIRIT) diagram. EQ-5D-5L five-level EuroQol-5D, PGIC Patients’ Global Impression of Change, SCS spinal cord stimulation

The study is designed as a superiority, parallel-group, three-centre RCT in patients with chronic neuropathic pain with a nested qualitative study and economic evaluation. Participants will be allocated in a 1:1 ratio to either a strategy of a screening trial followed by SCS implantation based on the screening trial result or an SCS implantation-only strategy. Both groups will be followed-up for 6 months post randomisation. Duration for the main study protocol (i.e. start of recruitment to last patient recruited completing all study procedures) is expected to be 26 months. Patients’ participation within the trial will last for 6 months following implantation. The study design is summarised in Fig. 1.

### Study population

One hundred adults (≥ 18 years) with chronic pain of neuropathic origin will be recruited from three centres: South Tees Hospitals NHS Foundation Trust (The James Cook University Hospital); Basildon and Thurrock University Hospitals NHS Foundation Trust; and Leeds Teaching Hospitals NHS Trust.

#### Inclusion criteria

- Adults (≥ 18 years old) who are clinically considered to be candidates for SCS as per NICE TA159 [15].
- Pain of neuropathic nature of an intensity of at least 5 as assessed on a numerical rating scale (NRS).
- Patient has persistent pain for more than 6 months despite appropriate conventional medical and surgical management including transcutaneous electric nerve stimulation (TENS), acupuncture, oral analgesic agents, cognitive behavioural therapy as well as nerve blockade where appropriate.
- Satisfactory multidisciplinary assessment by a team with expertise in delivering SCS therapy.
- Patient capable of providing informed consent.

#### Exclusion criteria

- Patient refusal to participate in the study.
- Presence of an ongoing pain condition considered by the investigator to overshadow the neuropathic pain condition to be treated with SCS.
- Current or previous treatment with an implanted pain relief device.
- Current participation or planned participation in other studies that may confound the results of this study.
- Ongoing anticoagulation therapy, which cannot be safely discontinued.
- Poor cognitive ability or lack of capacity.
- Unable to undergo study assessments or complete questionnaires independently.
- Patient is pregnant or planning to become pregnant during the course of the study.

#### Recruitment of patients

Patients will be recruited from the outpatient clinics of the three participating sites. Patients who are scheduled to have a spinal cord stimulator trial will be approached and given a Patient Information Sheet to take home to read. Informed consent will be obtained from suitable patients following a reasonable period of time by one of the Principal Investigators or delegated individuals at each site following International Conference on Harmonisation/Good Clinical Practice (ICH/GCP) guidelines [16].

### Interventions

#### Screening trial and implantation strategy (usual care)

Patients randomised to this arm will receive a screening trial. A screening trial will consist of passage of either an external or internalised tunnelled SCS lead or leads attached to an external stimulator as per the centre’s routine practice. Those patients who have a successful screening trial will receive an implantable neurostimulation system while unsuccessful patients would not receive such an implant. Taking into consideration the RCTs included in the clinical evidence section of NICE TA159 [15], a successful screening trial will be defined as ≥ 50% pain relief and satisfactory on-table paraesthesia coverage (i.e. ≥ 80%) of the pain area, reduction in pain medications or improved quality of life and function, and successful location of leads at the anatomical target for paraesthesia-free therapies [10, 17]. Patients with an unsuccessful screening trial will not be implanted but all patients will continue follow-up to 6 months. Successful trial patients will go on to have the implantable pulse generator (IPG) implanted on a separate occasion.

#### Implantation-only strategy

In the implantation-only strategy group, all patients with satisfactory on-table paraesthesia coverage (i.e. ≥ 80%) of the pain area and no dislike of sensations [18], and satisfactory anatomical lead location for paraesthesia-free devices, would receive a permanent implant in one surgery.

### Outcome measures

Patient outcomes will be collected at clinic visits at baseline (pre randomisation) and 6 months post randomisation unless otherwise stated.

#### Primary outcome

The primary outcome measure is the comparison between pain NRS at 6 months between the screening trial and implantation strategy and the implantation-only strategy [19].

#### Secondary outcomes

Secondary outcome measures include: the proportion of patients achieving at least 50% and 30% pain relief at 6 months as measured on the NRS [19], health-related quality of life (EQ-5D) [20], function (Oswestry Disability Index) [21], patient satisfaction (Patients’ Global Impression of Change) [22] and complication rates.

A qualitative study using semi-structured interviews will be carried out in parallel for a total of 30 of the patients recruited in each centre (10 at each centre) to explore their views of the screening trial, implantation and overall use of the SCS device.

Self-reported information on healthcare utilisation and non-NHS costs will be collected from participants using a standardised questionnaire to include management of adverse events (AEs), interventions, investigations, medication, inpatient hospitalisations, A&E, reprogramming visits and other healthcare-related visits, plus out-of-pocket costs and absences from work.

### Data collection and visits

Along with data collection at baseline, all of the outcomes will be sought through routine pain clinic attendances at 3 and 6 months. All unscheduled visits will be recorded in a log. The type of data collected during these visits is listed in Fig. 1 (SPIRIT diagram and Additional file 1).

#### Baseline visit

Patients who have been identified as potential participants will be sent a patient information sheet to read prior to the baseline visit. An MDT assessment will have been undertaken and the patient deemed suitable for SCS prior to the baseline visit. In the clinic, full written informed consent will be taken by the Principal Investigator or sub-investigator at the site or a suitable person as per the Delegation Log. Informed consent will be obtained during this visit.

#### Implant visit

Subjects who are randomised to the screening trial and implantation strategy will undergo two procedures as per usual clinical practice with a trial procedure and, if successful, a permanent implant in a second procedure. Details of the trial procedure will be as per usual clinical practice at the centre. Subjects who are randomised to the implantation-only strategy will undergo one procedure with the trial and permanent implant in one procedure. Wound care and management of the trial leads will be conducted as per the centre’s usual clinical practice regardless of trial randomisation.

#### One-month telephone interview following permanent implant (optional)

Subjects who have consented to undergo the qualitative interview and who have been randomised to do so will undergo a second interview.

#### Three-month and 6-month (end of study) visit following permanent implant

All patients will attend the clinic for the 3-month and 6-month appointments. Following the 6-month visit the patient will exit the study and be passed back to usual care within the pain clinic for ongoing management for the spinal cord stimulator.

#### Data collection and follow-up for withdrawn subjects

If a patient withdraws from the trial treatment, then they will be followed up wherever possible and data collected as per protocol until the end of the trial. The only exception to this is where the patient also explicitly withdraws consent for follow-up. Subjects who withdraw from the study will be initially invited to attend a follow-up appointment by letter. In the case of no response, the subject will be contacted by telephone twice over a 2-week period.

### Sample size determination

We plan to recruit and randomise a total of 100 patients (50 per group) in order to detect a statistically significant and clinically meaningful between-group difference using our primary outcome based on an intention-to-treat analysis. A sample size of 50 patients in the implantation strategy arm will determine our precision to estimate the specificity and sensitivity of the SCS screening test.

Assuming that the SCS screening trial has little or no clinical utility we would hypothesise superiority of the no-screening strategy over the screening strategy. For a pain NRS (0–10), IMMPACT proposes a minimal clinically important difference (MCID) of 2 points [19]. Based on a typical pain NRS standard deviation of 2.5 seen in previous SCS RCTs [10, 17, 23], at 90% power and 5% alpha, and a worst-case attrition rate of 30% [10, 17, 23], we will require a total of 50 patients recruited per group.

Given the lack of previously published sensitivity and specificity values for the SCS screening test, Table 1 presents our margins of error of estimation (width of the 95% confidence interval (CI)) based on 50 patients in the implantation strategy arm across a range of possible values of diagnostic performance.

**Table 1:** Margins of error of estimation based on 50 patients in the implantation strategy arm

Margin of error	Sensitivity^a^ (%)	Specificity^a^ (%)
100%	8.9	30.9
80%	26.6	53.3
60%	31.8	61.6
40%	31.8	61.6

^a^Assuming 40/50 patients have ≥ 50% pain relief at 6 months

### Randomisation

Randomisation will be achieved by means of a password-protected web-based system developed and maintained by Exeter Clinical Trials Unit (ExeCTU). Once the patient has completed the screening interview and baseline data collection interview, the researcher will access the randomisation website using a unique username and password. The website will require entry of the study site and participant age before returning the participants’ unique randomisation number and allocation (Engager Intervention or Control). Allocation will be stratified by centre and minimised on patient age (≥ 65 or < 65 years), gender, presence of FBSS or not and use of high frequency (HF10™) or not. Allocation concealment will be maintained by only revealing allocation of each participant to the study manager following completion of written informed consent and baseline outcomes.

It is not possible to blind patients, clinicians or researchers to group allocation. However, to minimise assessment bias we will seek to blind the researchers undertaking outcome assessment and the data analysts to group allocation by masking them from group allocation. Each site team consists of a blinded and an unblinded assessor. These do not cross roles or exchange information. Database entries are also clearly divided into blinded and unblinded sections with no potential for crossed data entry since blinded assessor login only allows access to a limited set of data.

All sites have clinical experience with both SCS implantation after the extended trial and immediate SCS implantation after the on-table trial. Research and clinical teams are equipoised as regards the outcome of this clinical trial.

### Statistical analysis

#### Diagnostic performance

Analyses will be conducted and reported in accord with STARD recommendations [24]. Cross-tabulation will be used to report the SCS test results (fail versus pass) versus SCS pain relief (≥ 50% versus < 50%) at 6-month follow-up. Sensitivity will be determined as the percentage of participants with ≥ 50% pain relief at 6 months who pass the test, and specificity as the percentage of participants with < 50% pain relief who fail the test. Positive and negative likelihood ratios will be also calculated and reported. All results will be reported with 95% CIs.

#### Comparison of effectiveness

**Fig. 2:**
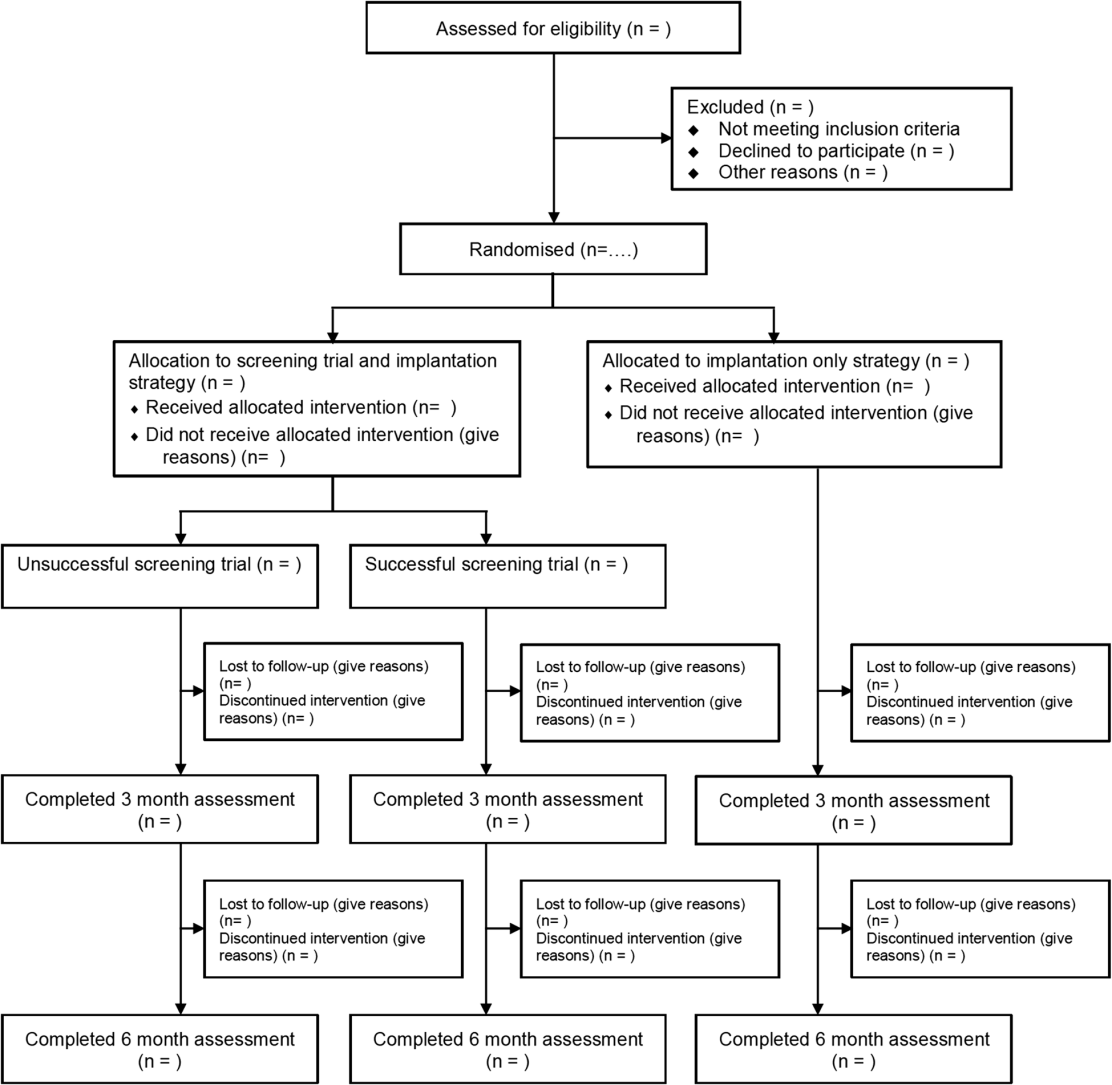
Consolidated Standards Of Reporting Trials (CONSORT) flow diagram

Analyses will be conducted and reported in accord with CONSORT recommendations [25]. We will closely monitor the process of data collection during the trial and provide a flow diagram summarising, by group, the numbers approached, recruited, randomised, followed up/lost to follow-up and outcome completion (Fig. 2). Primary analyses will be conducted on an intention-to-treat basis (i.e. according to randomised group) and to compare primary and secondary outcomes at 6-month follow-up between randomised groups on those with complete datasets. Outcomes will be compared using linear regression methods adjusting for baseline outcome scores and stratification/minimisation variables. Additional secondary analyses will be performed. Primary and secondary outcomes will be compared at 3 months as already described. The influence of missing data will be investigated using sensitivity analyses that make different assumptions, such as “best” and “worst” case scenarios depending on outcome type, as well as using multiple imputation methods. Exploratory analyses using interaction terms will be used to assess the potential subgroup effects according to stratification and minimisation variables.

No interim or subgroup analyses are planned. All analyses will be undertaken using STATA v.14. A detailed statistical analysis plan will be prepared before any data analysis is conducted and agreed with the Trial Steering Committee (TSC)/Trial Management Group (TMG).

The study team will have access to the final trial dataset.

### Economic evaluation

An economic evaluation will be carried out to estimate the cost-effectiveness of a screening trial and implantation strategy versus an implantation-only strategy. Healthcare resource utilisation (e.g. management of AEs, interventions, investigations, medication, inpatient hospitalisations, A&E and other healthcare-related visits, plus out-of-pocket costs and absences from work) will be collected for each patient during the study follow-up period using data collection methods which have been successfully adopted by the applicants in previous studies [10, 23]. Resources required for the specific screening trial and implantation interventions will be recorded within the trial.

Items of resource use will be costed using national averages obtained from national sources (such as the Personal Social Services Research Unit, the British National Formulary and NHS reference cost databases) [26–28]. Cost components will be combined to derive total patient-level costs for the NHS. In addition, non-NHS costs such as productivity loss due to absence from work or patient out-of-pocket expenses will also be quantified to provide a full picture of how the strategies being compared will affect the financial burden imposed by the condition on both the NHS and the patients. Generic health-related quality of life (HRQoL) data will be collected using the EQ-5D-5L instrument. Both resource utilisation (costs) and the EQ-5D-5L will be collected at each follow-up visit. A within-trial cost consequence analysis will be carried out to estimate mean resource utilisation, costs, EQ-5D scores and total quality-adjusted life-years (QALYs) in each group, together with relevant measures of sampling uncertainty. QALYs will be calculated using the area under the curve approach, with regression-based adjustment for baseline EQ-5D score. The economic evaluation will take the form of a cost–utility analysis, to calculate the cost per additional QALY gained. Base-case analyses will be conducted from the NHS perspective, with additional analyses from the societal perspective. Deterministic sensitivity analysis will be undertaken to explore the robustness of the results to plausible variations in key assumptions and variations in the analytical methods used. In order to account for uncertainty, probabilistic sensitivity analysis (PSA) will also be undertaken using bootstrapping and the bootstrapped cost–effect pairs will be graphically presented on a cost-effectiveness plane. Cost-effectiveness acceptability curves will be constructed to show the probability that the intervention is cost-effective for specific thresholds of cost per QALY gained.

A two-stage economic model will also be developed with a decision tree reflecting the outcomes of the first 6 months and reproducing the observed TRIAL-STIM Study outcomes. After this 6-month period, a Markov “state transition” model will then extrapolate the evolution of patient outcome and costs over a period of 15 years. The model will be populated with data from the trial itself and previously published research. Again, extensive deterministic and probabilistic sensitivity analysis will be undertaken.

### Qualitative analysis

All subjects will be consented to take part in both the RCT and the optional qualitative study at the outset. A semi-structured telephone interview will be carried out with 30 patients recruited by purposive sampling (among those consenting) at three sites (at baseline and 1 month post implantation). Baseline interviews will explore the impact of pain on daily living, thoughts and understanding of the screening trial and implantation only and expectations of the spinal cord stimulator. One-month follow-up interviews will revisit thoughts and understanding of the spinal cord stimulators, expectations and explore their overall experience of the SCS device. Ten patients will be recruited from each centre, five from each treatment arm. Open-ended questions will be used to allow patients to express their own viewpoints. The interviews will be audio recorded and transcribed. Data will be analysed using thematic analysis [29, 30], facilitated by NVivo qualitative analysis software. The data will be interpreted as follows:

1. themes emerging for the total patient group; and
2. themes emerging for the patients grouped by arm of the study (screening trial/implantation only).

In addition, participants will be asked to reflect and explore their experience of being in the study and evaluate things that went well, as well as to provide suggestions for changes and to complete a purpose-designed 5-point scale indicating their overall preference in relation to the two options (screening trial/implantation only).

### Trial management and quality assurance

#### Trial Steering Committee

The trial is guided by a group of respected and experienced pain consultants as well as research personnel. The steering committee is chaired by Dr Richard North (RN).

The membership of the groups will consist of the Chief Investigator (SE), RN as an independent member, study statistician (RST), independent statistician (Professor Alan Batterham, AB), health economics study team (SJ and RD), psychologist representing qualitative interviews (RC), study manager (MB) and a Patient and Public Involvement (PPI) representative as a lay person (JE).

Video/teleconference meetings will be held at regular intervals determined by need but not less than twice a year. Routine business is conducted by email, post or teleconferencing.

The Steering Committee, in the development of this protocol and throughout the trial, will take responsibility for:

- major decisions such as a need to change the protocol for any reason;
- monitoring and supervising the progress of the trial;
- reviewing relevant information from other sources;
- reviewing data management and ethical arrangements; and
- informing and advising on all aspects of the trial.

#### Trial Management Group

The administration will be overseen by a TMG chaired by the Chief Investigator.

Membership of the TMG will include the principal investigator (PI) on all three sites (AG, ST and GB), members of the qualitative studies team (RC/HS), members of the health economics team (RD/SJ), study statistician (RST), study manager (MB) and study nurses from all study sites.

The TMG will meet at monthly intervals initially at set up and then 3-monthly either face to face or by tele/videoconferencing where possible.

The trial and day-to-day, non-clinical aspects will be coordinated by the Study Manager (MB) based at The James Cook University Hospital. All clinical coordination of the trial will be the responsibility of the Chief Investigator. The Chief Investigator will assume responsibility for the overall management and conduct of the trial, and AG, ST and GB will act as PI for the Middlesbrough, Basildon and Leeds sites respectively. Each PI will assume responsibility for leading the trial in their centre.

The trial office team will:

- distribute the case report forms (CRFs) to participating centres;
- monitor the collection of data, process data, seek missing data and clarify ambiguous data;
- ensure the confidentiality and security of all trial forms and data;
- liaise with Exeter CTU regularly regarding data entry and randomisation;
- coordinate any interim and main analyses; and
- organise Steering Committee and Collaborator meetings.

The trial office will receive and check completed CRFs via the postal service as well as the online database. Upon receipt, data forms will be checked for completeness and a sample checked against the online study database. Data will be downloaded onto a master chart, which will be communicated to the study statistician.

Patient confidentiality will be maintained at every stage and we will comply with the Data Protection Act (1998).

Any further amendments to the protocol will be submitted to a properly constituted Research Ethics Committee (REC) for approval of the study conduct.

### Safety monitoring

An AE is defined as any untoward medical occurrence in a patient during or following administration of an investigational product/procedure and which does not necessarily have a causal relationship with treatment. An AE can therefore be any unfavourable and unintended sign (including an abnormal laboratory finding), symptom or disease temporarily associated with the use of the trial device, whether or not considered related to the trial device. PIs will assess all AEs regarding their relationship to SCS. The SCS-related AEs include but are not limited to: occurrence of redness, tenderness or other sign of infection over the SCS implant site; observation of collections or other abnormalities over implant sites; shifting of the site of paraesthesia from the site originally intended, causing reduction in pain relief with inability to shift paraesthesia to the original position using programming; lead migration, either vertical or mediolateral; pain reports over implant sites; and patient reports of cessation of paraesthesia not remedied by reprogramming of the device. All adverse events will be followed until they have abated, or until a stable situation has been reached. Depending on the event, follow-up may require additional tests or medical procedures as indicated, and/or referral to the general physician or a medical specialist.

All clearly related signs, symptoms and abnormal diagnostic procedures or results will be recorded in the source document and grouped under one diagnosis. All adverse events occurring during the study period will be recorded. The independent members of the Steering Committee will be presented with the safety data prior to the Steering Group Meetings.

The trial is subject to the audit arrangements of the National Institute of Health Research (NIHR).

### Dissemination policy

For primary result publications, the study team will form the basis of the writing committee and will also advise on any related publications from the trial. Primary publications will include co-investigators as named authors or as part of a group authorship. In general, any related publications should include the principal investigators, lead researchers, statisticians and site staff as named authors; however, this is at the discretion of the writing committee.

## Discussion

SCS has been tested against various control therapies in the management of neuropathic pain of FBSS as well as painful diabetic neuropathy and Complex Regional Pain Syndrome (CRPS) [10, 17, 31]. All studies have implemented a screening trial prior to implantation of the final permanent components of the therapy. While generally recommended by most guidelines [15, 32], none of the guidelines present an evidence-based rationale for screening trials.

### Pros and cons of screening trials

Screening trials give patients the opportunity for a short period of experiencing SCS therapy and allow them to experience the sensation generated by SCS and its interaction with body movements. This also allows patients and physicians a baseline evaluation of the pain relief and current consumption, which influence the choice of battery implanted. However, screening trials require duplication of procedures, thereby consuming more healthcare resources. In a recent RfPB-funded study the applicants adopted a no-screening trial policy with average on-table total implant time of 111 min [23]. By contrast, the normal screening trial surgery duration averages 90 min and pulse generator implant surgery averages 60 min [33].

Moreover, prolonged SCS screening trials expose patients to a higher risk of infection due to bacterial colonisation of the wire skin exit site. Longer trials of up to 15 days have been associated with an infection rate of 7.5% compared to an average infection rate in the same department of 2.8% [8]. As most SCS infections require device removal this is an expensive as well as an inconvenient complication. We also found screening trials to be associated with severe and prolonged surgical pain, calling into question a patient’s ability to judge the impact of SCS on their original pain.

In conclusion, published studies to date call into question the predictive value of screening trials. While screening trials allow patients to experience first-hand the effects of SCS therapy before deciding to receive a permanent implant, screening trials are costly, require procedure duplication, are associated with increased risk of infection and may not appropriately represent therapeutic long-term outcomes. The current NICE recommendation [15] that all candidates for SCS undergo screening trials is largely based on expert opinion rather than firm evidence.

A number of studies have examined the predictive value of screening trials in predicting long-term outcome of SCS therapy, but none with an appropriate design or adequate power [14, 34].

Weinand et al. [14] conducted a retrospective case series of 54 patients. All patients had an acute 15-min on-table trial followed by an externalised approximately 5-day trial with a mean follow-up of 9.4 ± 1.5 months. The screening trial success rate was reported at 98% in acute screening and 90% in prolonged screening. The authors concluded that acute and prolonged SCS screening appear to have equivalent predictive value for successful long-term SCS control of chronic low back and/or lower extremity pain. These preliminary results suggest potential justification for eliminating prolonged and retaining acute (intraoperative) SCS screening for selection of permanent SCS implantation candidates. The investigators found no clear difference between acute on-table trials and prolonged outpatient trials and concluded that on-table acute trials were more cost-effective. Moriyama et al. [34] conducted a prospective cohort of 55 patients of various pain aetiologies implanted with SCS and followed up for 6 months. They reported a screening trials success rate of 61.8% (34/55), reported the factors predicting a positive response to include increased paraesthesia coverage, female gender and disorders of peripheral vs central nervous system, and reported screening trial sensitivity of 84.8% and specificity of 70% (14/20).

Given that both of these studies reported such a wide margin of screening trial success and included a small sample size, we believe that the proposed TRIAL-STIM Study is fully justified clinically and economically.

### Trial status

The TRIAL-STIM Study began recruitment in June 2017. The trial is scheduled to end recruitment in January 2019.

## Additional file

Additional file 1: SPIRIT checklist. (DOC 135 kb)

## Abbreviations

FBSS: Failed back surgery syndrome
ICH/GCP: International Conference on Harmonisation/Good Clinical Practice
MCID: Minimal clinically important difference
NHS: National Health Service
NIHR: National Institute for Health Research
NRS: Numerical rating scale
PPI: Patient and public involvement
RCT: Randomised controlled trial
RfPB: Research for patient benefit
SCS: Spinal cord stimulation
TMG: Trial Management Group
TSC: Trial Steering Committee

## References

[1] Bouhassira D (2008). Prevalence of chronic pain with neuropathic characteristics in the general population. Pain.

[2] Breivik H (2006). Survey of chronic pain in Europe: prevalence, impact on daily life, and treatment. Eur J Pain.

[3] Torrence N (2006). The epidemiology of chronic pain of predominantly neuropathic origin. Results from a general population survey. J Pain.

[4] Smith BH (2007). Health and quality of life associated with chronic pain of predominantly neuropathic origin in the community. Clin J Pain.

[5] Torrance N, Smith BH, Lee AJ (2009). Analyzing the SF-36 in population-based research. A comparison of methods of statistical approaches using chronic pain as an example. J Eval Clin Pract.

[6] Galvez R (2009). Varaiable use of opioid pharmacotherapy fro chronic noncancer pain in Europe: causes and consequences. J Pain Palliat Care Pharmacother.

[7] Simpson EL (2009). Spinal cord stimulation for chronic pain of neuropathic or ischaemic origin: systematic review and economic evaluation. Health Technol Assess.

[8] Chincholkar M (2011). Prospective analysis of the trial period for spinal cord stimulation treatment for chronic pain. Neuromodulation: Technology at the Neural Interface.

[9] Deer TR (2014). The appropriate use of neurostimulation of the spinal cord and peripheral nervous system for the treatment of chronic pain and ischemic diseases: the Neuromodulation Appropriateness Consensus Committee. Neuromodulation.

[10] Kumar K (2007). Spinal cord stimulation versus conventional medical management for neuropathic pain: a multicentre randomised controlled trial in patients with failed back surgery syndrome. Pain.

[11] North RB (2005). Spinal cord stimulation versus repeated lumbosacral spine surgery for chronic pain: a randomised, controlled trial. Neurosurgery.

[12] Kapural L (2015). Novel 10-kHz high-frequency therapy (HF-10 Therapy) is superior to traditional low-frequency spinal cord stimulation for the treatment of chronic back and leg pain: the SENZA-RCT randomized controlled trial. Anesthesiology.

[13] Oakley JC (2008). Successful long-term outcomes of spinal cord stimulation despite limited pain relief during temporary trialing. Neuromodulation.

[14] Weinand ME (2003). Acute vs. prolonged screening for spinal cord stimulation in chronic pain. Neuromodulation.

[15] NICE (2008). NICE Technology Appraisal Guidance 159: Spinal cord stimulation for chronic pain of neuropathic or ischaemic origin.

[16] NIHR (2011). A Pocket Guide to Good Clinical Practice, Including the Declaration of Helsinki. Vol. Version 2.1.

[17] Kemler MA (2000). Spinal cord stimulation in patients with chronic reflex sympathetic dystrophy. N Engl J Med.

[18] Eldabe S (2013). The Effectiveness and Cost-Effectiveness of Spinal Cord Stimulation for Refractory Angina (RASCAL study): study protocol for a pilot randomized controlled trial. Trials.

[19] Dworkin RH (2005). Core outcome measures for chronic pain clinical trials: IMMPACT recommendations. Pain.

[20] Rabin R, de Charro F (2001). EQ-5D: a measure of health status from the EuroQol Group. Ann Med.

[21] Fairbank JC (1980). The Oswestry low back pain disability questionnaire. Physiotherapy.

[22] Guy W (1976). Clinical global impressions.

[23] Eldabe S (2016). The Effectiveness and Cost-Effectiveness of Spinal Cord Stimulation for Refractory Angina (RASCAL Study): a pilot randomized controlled study. Neuromodulation: Technology at the Neural Interface.

[24] Bossuyt PM (2015). An updated list of essential items for reporting diagnostic accuracy studies. BMJ.

[25] Schultz KF, Altman DG, Moher D (2010). CONSORT 2010 Statement: updated guidelines for reporting parallel group randomised trials. BMJ.

[26] Joint Formulary Committee (2018). British National Formulary.

[27] Department of Health. NHS Reference Costs 2015/2016. Available from: https://www.gov.uk/government/publications/nhs-reference-costs-2015-to-2016.

[28] Curtis L, Burns A (2017). Unit costs of health and social care. Personal Social Services Research Unit.

[29] Fereday J, Muir-Cochrance E (2006). Demonstrating rigor using thematic analysis: a hybrid approach of inductive and deductive coding and theme development. Int J Qual Methods.

[30] Joffe H, Harper D, Thompson AR (2011). Thematic analysis. Qualitative research methods in mental health and psychotherapy: a guide for students and practitioners.

[31] Slangen R (2014). Spinal cord stimulation and pain relief in painful diabetic peripheral neuropathy: a prospective two-center randomized controlled trial. Diabetes Care.

[32] Deer TR (2017). The Neurostimulation Appropriateness Consensus Committee (NACC) Safety Guidelines for the Reduction of Severe Neurological Injury. Neuromodulation.

[33] Manca A (2008). Quality of life, resource consumption and costs of spinal cord stimulation versus conventional medical management in neuropathic pain patients with failed back surgery syndrome (PROCESS trial). Eur J Pain.

[34] Moriyama K (2012). A prospective, open-label, multicenter study to assess the efficacy of spinal cord stimulation and identify patients who would benefit. Neuromodulation: Technology at the Neural Interface.

