# Correction to: Does a Screening Trial for Spinal Cord Stimulation in Patients with Chronic Pain of Neuropathic Origin have Clinical Utility and Cost-Effectiveness? (TRIAL-STIM Study): study protocol for a randomised controlled trial

**DOI:** 10.1186/s13063-019-3804-7

**Published:** 2019-10-28

**Authors:** Sam Eldabe, Ashish Gulve, Simon Thomson, Ganesan Baranidharan, Rui Duarte, Susan Jowett, Harbinder Sandhu, Raymond Chadwick, Morag Brookes, Anisah Tariq, Jenny Earle, Jill Bell, Anu Kansal, Shelley Rhodes, Rod S. Taylor

**Affiliations:** 1 The James Cook University Hospital, Middlesbrough, UK; 2 Basildon and Thurrock University Hospitals, Basildon, UK; 3 Leeds Teaching Hospitals, Leeds, UK; 4 University of Liverpool, Liverpool, UK; 5 University of Birmingham, Birmingham, UK; 6 University of Warwick, Warwick, UK; 7 University of Teesside, Middlesbrough, UK; 8 Patient and Public Involvement Representatives, Middlesbrough, UK; 9 University of Exeter, Exeter, UK

**Correction to: Trials (2018) 19: 633**

**https://doi.org/10.1186/s13063-018-2993-9**

Following publication of the original article [1], we have been notified that the final specification of randomisation implemented in the study is slightly different to that stated in the protocol and needs to be corrected as follows:

- Allocation will be stratified by centre and minimised on patient age (≥ 65 or < 65 years), gender, **and** presence of FBSS or not
